# Selection of species specific panel of reference genes in peripheral blood mononuclear cells of native livestock species adapted to trans-Himalayan region of Leh-Ladakh

**DOI:** 10.1038/s41598-022-22588-0

**Published:** 2022-11-02

**Authors:** Manish Tiwari, Monika Sodhi, Preeti Verma, Prince Vivek, Ranjit S. Kataria, Saket K. Niranjan, Vijay K. Bharti, Nampher Masharing, Gayatri Gujar, Divya Chanda, Manishi Mukesh

**Affiliations:** 1 Animal Biotechnology Center, ICAR-National Bureau of Animal Genetic Resources, Karnal, Haryana India; 2 DRDO-Defense Institute of High-Altitude Research, Leh, India

**Keywords:** Biological techniques, Gene expression analysis, Reverse transcription polymerase chain reaction

## Abstract

The identification of appropriate references genes is an integral component of any gene expression-based study for getting accuracy and reliability in data interpretation. In this study, we evaluated the expression stability of 10 candidate reference genes (*GAPDH, RPL4, EEF1A1, RPS9, HPRT1, UXT, RPS23, B2M, RPS15, ACTB*) in peripheral blood mononuclear cells of livestock species that are adapted to high altitude hypoxia conditions of Leh-Ladakh. A total of 37 PBMCs samples from six native livestock species of Leh-Ladakh region such as Ladakhi cattle, Ladakhi yak, Ladakhi donkey, Chanthangi goat, Double hump cattle and Zanskar ponies were included in this study. The commonly used statistical algorithms such as geNorm, Normfinder, BestKeeper and RefFinder were employed to assess the stability of these RGs in all the livestock species. Our study has identified different panel of reference genes in each species; for example, *EEF1A1*, *RPL4* in Ladakhi cattle*; GAPDH, RPS9*, *ACTB* in Ladakhi yak; *HPRT1*, *B2M, ACTB* in Ladakhi donkey; *HPRT1*, *B2M, ACTB* in Double hump camel*, RPS9*, *HPRT1* in Changthangi goat, *HPRT1* and *ACTB* in Zanskar ponies. To the best of our knowledge, this is the first systematic attempt to identify panel of RGs across different livestock species types adapted to high altitude hypoxia conditions. In future, the findings of the present study would be quite helpful in conducting any transcriptional studies to understand the molecular basis of high altitude adaptation of native livestock population of Leh-Ladakh.

## Introduction

In recent years, high‐throughput techniques such as serial analysis of gene expression (SAGE), expressed sequence tag (EST), microarray and RNA-seq have been widely employed to study the gene functions and understand the transcriptional regulations in humans, animals as well as plants^1–5^. However, the high throughput expression data requires validation using real-time quantitative polymerase chain reaction (qPCR). The qPCR technique because of its dynamic range, scalability, sensitivity, and reproducibility has always been considered as precise technique to estimate the relative abundance of mRNA transcripts in any cell types^6–9^. However, in order to perform appropriate gene expression analysis, it has become mandatory to select stable reference genes (RGs) that can normalize provide accurate and reliable qPCR results for each and every experimental condition^10^. Earlier, most of the studies included single traditionally used housekeeping genes such as *GAPDH* or *ACTB* which might not provide accurate normalization of expression data. Later on, it has been strongly advocated that panel of two or more RGs should be employed for normalizing the expression data of target genes^11,12^. Further, the choice of appropriate RGs for individual experimentation dealing with varied biological resources was also realized for achieving the reproducibility and correct inferences^13^. Since last one decade or so, there has been significant increase in use of RT-qPCR technique to perform gene expression studies in different livestock and poultry species. Unfortunately, many of the studies didn’t follow the correct procedures to normalize the gene expression data^13^ Therefore, concerns have been raised by researchers across the world to ensure accuracy, consistency and reliability of RT-qPCR data by employing proper normalization methods.

Identification of panel of RGs has become the most popular approach to normalize the qPCR-based gene expression data as evident from numerous publications across mouse^14–16^, human^5,17^ plants^18,19^ and livestock species^20–24^. Lack of appropriate RGs can greatly compromise the reliability of qPCR due to technical variations or errors arises during sample preparation, like quality and starting amount of RNA, efficiency of reverse transcription, efficiency of PCR and errors during pipetting^25^. All these technical variations will affect both the target genes as well as selected panel of RGs. Therefore, it’s important to normalize the gene expression data by identifying suitable RGs or internal control genes (ICGs) in order to obtain an accurate and reliable gene expression data. Identification and validation of appropriate RGs has thus become an essential component in any gene expression studies wherein RGs are exposed to the same experimental conditions as target genes^26^.

Ladakh, one of the world’s highest inhabited region (3500–5500 m above sea level),  home to several unique native animal genetic resources such as cattle, yak, goat, sheep, donkeys, horses and double hump camel. The economy of local people is mainly dependent on these livestock species. The native cattle known as “Ladakhi cattle” (*Bos indicus*) is a unique germplasm that provides 2.5–4.5 kg of milk and has excellent adaptation potential to high altitude hypobaric hypoxia stress^27,28^. The Ladakhi yak (*Bos grunniens*) is also very well adapted and major source of milk and milk products for the local people. The Ladakhi goat (*Capra hircus*) or world famous pashmina goat is mainly reared for meat, milk, fiber (Pashmina and Mohair), hide and skin. Ladakhi donkey (*Equus asinus*) and Zanskari ponies (*Equus caballus*) are yet another important animal genetic resources that serves as an important pack animal for the local people and Indian army. Another unique species; double hump camel (*Camelus bactrianus***)** is quite popular amongst tourists especially for safari in world famous cold desert stretch of Nubra valley region of Ladakh. Each of these species has developed effective mechanism to survive at high altitude and low oxygen condition. Under such adverse climatic conditions, the survival and performance of exotic breeds is not a viable option. It only allows the well adapted animal genetic resources to thrive and perform. Therefore, understanding transcriptome signatures and identifying genes highly abundant across all these species will provide strong clue on molecular mechanism operating at transcriptional level in response to abiotic hypoxia stress across these species. By making such advancements, not only these resources will be characterized and documented but will also help to understand these unique animals production attribute in a better way for future exploitation and overall improvement. As a step forward, the present study was designed to identify and select panel of stably expressed RGs for future transcriptional studies in each of the six livestock species of Ladakh.

In recent past, numerous studies have been conducted in similar lines to identify panel of appropriate RGs in several livestock species such as cattle^22,29^ buffaloes^20,21^, yak^30^, pig^31^, goat^32,33^ sheep^33,34^, horse^35^ etc*.* These studies have represented wide array of environmental or experimental conditions such as responses to external stimuli (heat stress, endurance, exercise), physiological or developmental stages, lactation cycle, cellular response etc*.*^20–22,28,30,35–37^. It is now evident that set of RGs that perform well in one particular condition or species may not work well in other experimental conditions or other species. Therefore, in the present study, an effort was made to evaluate and identify panel of appropriate RGs in peripheral blood mononuclear cells (PBMCs) of six livestock species well adapted to high altitude region of Leh-Ladakh viz., Ladakhi cattle, Ladakhi yak, Ladakhi donkey, Changthangi goat, Zanskar ponies and double hump camel. All these livestock species are native of Leh and Ladakh and have been naturally selected not only to sustain but perform and reproduce well under high altitude hypoxia stressful conditions. The 10 candidate RGs that were evaluated in the present study were; glyceraldehyde 3-phosphate dehydrogenase (*GAPDH*), beta-Actin (*ACTB*), ubiquitously expressed transcript (*UXT*), ribosomal protein S15A (*RPS15A*), beta 2-microglobulin (*B2M*), ribosomal protein L-4 (*RPL4*), ribosomal proteinS18 (*RPS18*), ribosomal protein S9 *(RPS9*), ribosomal protein S23 (*RPS23*), hydroxymethylbilane synthase (*HMBS*), hypoxanthine phosphoribosyl transferase (*HPRT1*).

## Results

### Specificity, expression abundance and coefficient of variation of individual RGs

**Figure 1:**
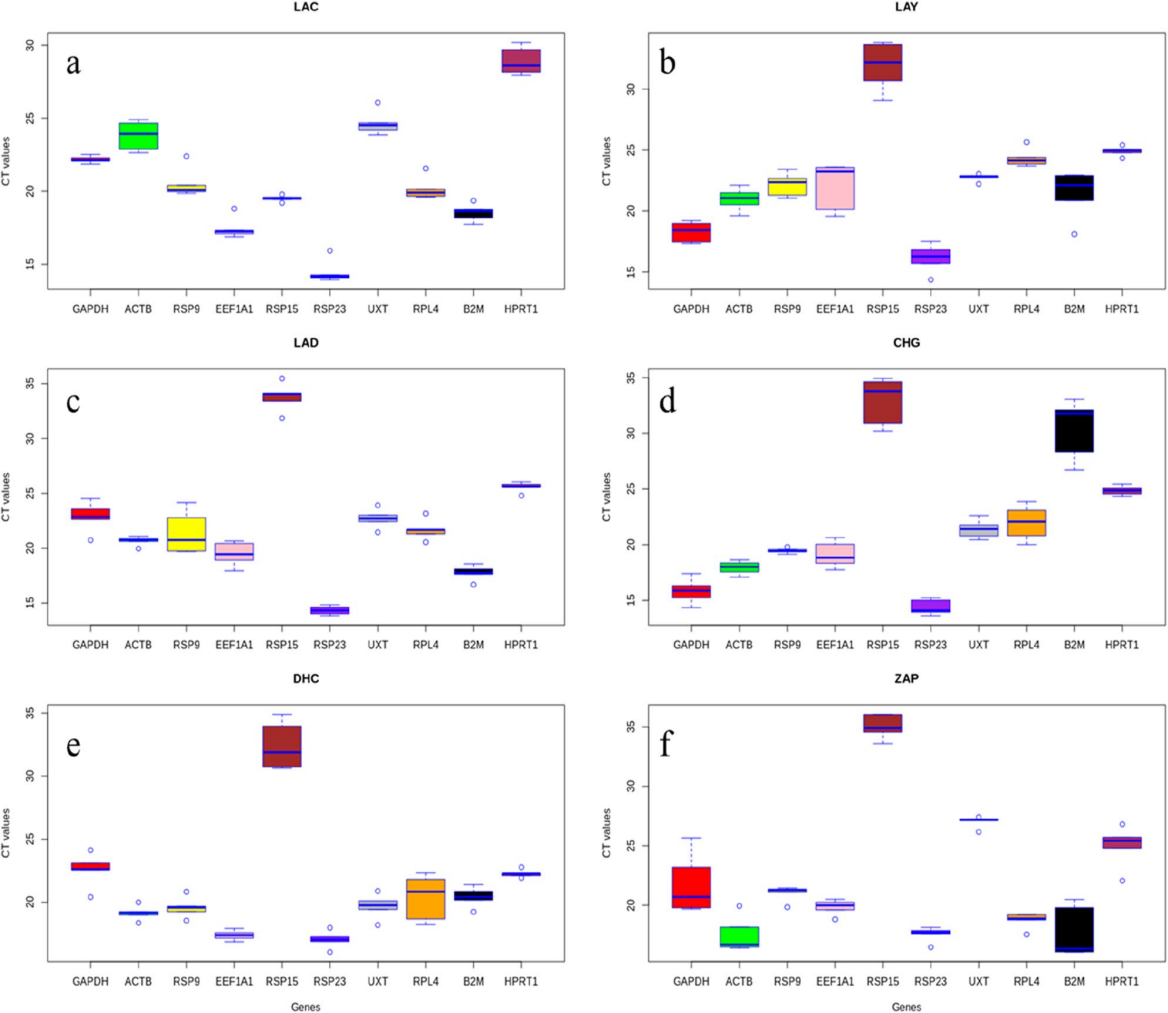
Expression level of individual candidate RGs in LAC (**a**), LAY (**b**), LAD (**c**), CHG (**d**), DHC (**e**) and ZAP (**f**). The data is presented as quantification cycle (Ct) values of each gene in the box-whisker diagram. The median is shown as a line across the box while whiskers indicate maximum and minimum values.

In the present study, an effort was made to identify the appropriate RGs in all the major livestock species that are native of Leh-Ladakh region viz., Ladakhi cattle (LAC), Ladakhi yak (LAY), Ladakhi donkey (LAD), Changthangi goat (CHG), Double hump camel (DHC), Zanskar ponies (ZAP). The specificity of each primer pair was confirmed by the specific amplification checked in agarose gel and presence of single peak in melt curve analysis. The correlation coefficient (R^2^) and amplification efficiency (E) for individual primer pair in each of the six livestock species are given in Table 1. The expression abundance of individual RGs in each species is shown in Box Whisker plot (Fig. 1a–f). The Ct values of individual RGs ranged from *RPS23* (13.94) to *HPRT1* (30.18) in LAC; *RPS23* (14.37) to *RPS15* (33.82) in LAY; *RPS23* (13.86) to *RPS15* (35.47) in LAD; *RPS15* (16.05) to *RPS23* (34.90) in DHC; *RPS15* (13.63) to *RPS23* (34.92) in CHG; *RPS15* (16.06) to *RPS23* (36.06) in ZAP (Table 2).

**Table 1:** Gene symbol, accession number, primer sequence, melting temperature (T_a_), amplicon size, slope, PCR efficiency and R^2^ of RGs for each evaluated RG.

Gene symbol	Accession number	Primers 5′–3′ (forward, reverse)	T_a_ (°C)	Amplicon size (bp)	Slope	PCR efficiency	R^2^
Beta-Actin (*ACTB*)	NM_173979.3	F:5′GCGTGGCTACAGCTTCACC3′ R:3′TTGATGTCACGGACGATTTC5′	60	56	− 3.10	107.40	0.997
Glyceraldehyde 3-phosphate dehydrogenase (*GAPDH*)	NM_001034034.2	F:5′TGGAAAGGCCATCACCATT3′ R:3′CCCACTTGATGTTGGCAG5′	60	60	− 2.99	119.28	0.997
Eukaryotic translation elongation factor 1 alpha 1(*EEF1A1*)	NM_174535.2	F:5′CATCCCAGGCTGACTGTGC3′ R:3′TGTAAGCCAAAAGGGCATG5′	60	101	− 3.11	109.65	0.998
β2 Microglobulin (*B2M*)	XM_002691119.4	F:5′CTGCTATGTGTATGGGTTCC3′ R:3′GGAGTGAACTCAGCGTG5′	60	101	− 3.03	114.64	0.999
Ribosomal protein L4 (*RPL4*)	NM_001014894.1	F:5′TTGGAAACATGTGTCGTGG3′ R:3′GCAGATGGCGTATCGCTTCT5′	60	101	− 3.12	109.45	0.998
Ribosomal protein S15 (*RPS15*)	NM_001037443.2	F:5′GAATGGTGCGCATGAATGT3′ R:3′GACTTTGGAGCACGGCCTA5′	60	101	− 2.89	127.12	0.996
Ribosomal protein S23 (*RPS23*)	NM_001034690.2	F:5′CCCAATGATGGTTGCTTGAA3′ R:3′CGGACTCCAGGAATGTCAC5′	60	101	− 3.20	102.27	0.990
Ribosomal protein S9 (*RPS9*)	NM_001101152.2	F:5′CCTCGACCAAGAGCTGAAG3′ R:3′CCTCCAGACCTCACGTTTGT5′	60	54	− 3.03	113.54	0.996
Ubiquitously expressed transcript (*UXT*)	NM_001037471.2	F:5′TGTGGCCCTTGGATATGGTT3′ R:3′GGTTGTCGCTGAGCTCTGTG5′	60	101	− 3.33	99.36	0.988
Hypoxanthine Phosphoribosyl transferase (*HPRT1*)	NM_001034035.2	F:5′GAGAAGTCCGAGTTGAGTT3′ R:3′GGCTCGTAGTGCAAATGAA5′	60	101	− 3.03	113.60	0.988

**Table 2:** The average raw Ct values of individual RGs in different species.

S. no.	SAMPLE	*GAPDH*	*ACTB*	*RPS9*	*EEF1A1*	*RPS15*	*RPS23*	*UXT*	*RPL4*	*B2M*	*HPRT1*
1	LAC 1	22.166	24.350	22.395	18.810	19.511	15.934	26.065	21.560	19.355	30.175
2	LAC 2	21.865	22.880	20.150	17.320	19.785	14.275	23.860	20.055	18.195	28.375
3	LAC 3	22.080	23.515	19.975	16.895	19.550	14.240	24.175	19.665	18.715	27.950
4	LAC 4	22.295	24.660	19.865	17.260	19.570	14.094	24.545	20.145	18.565	28.135
5	LAC 5	22.525	22.640	20.035	17.090	19.205	13.943	24.675	19.605	18.745	28.865
6	LAC 6	22.065	24.900	20.410	17.225	19.445	14.062	24.505	19.750	17.735	29.690
7	LAY 11	18.984	22.124	22.675	23.616	33.537	17.509	22.220	23.867	22.913	24.790
8	LAY 12	17.341	20.518	21.299	20.124	29.057	15.891	22.872	24.407	20.860	24.951
9	LAY 15	17.458	19.617	21.063	19.573	30.821	14.365	22.848	25.646	18.109	24.333
10	LAY 16	18.306	21.148	22.322	23.314	30.667	15.700	23.041	23.709	22.436	25.037
11	LAY 18	19.202	21.497	23.420	23.584	33.815	16.677	22.838	24.405	22.954	25.410
12	LAY 19	18.548	20.981	22.407	23.161	33.664	16.822	22.763	23.866	21.809	25.026
13	LAD 1	22.631	21.085	19.703	18.913	34.072	14.334	23.918	21.676	17.632	25.613
14	LAD 2	24.527	20.870	20.745	20.654	31.857	14.842	22.433	21.309	18.578	26.040
15	LAD 3	20.735	20.623	19.752	17.940	33.395	14.630	23.017	23.167	16.685	24.795
16	LAD 4	23.579	19.967	22.779	19.457	34.096	14.027	22.706	21.676	18.105	25.826
17	LAD 5	22.868	20.767	24.162	20.442	35.473	13.857	21.455	20.553	17.750	25.568
18	CHG 27	14.350	18.640	19.792	18.836	34.923	13.633	21.406	23.870	28.316	25.075
19	CHG 28	15.877	18.359	19.575	20.644	30.897	15.230	20.443	20.810	33.046	25.448
20	CHG 29	17.404	17.562	19.358	18.338	34.623	15.027	22.621	23.095	32.082	24.355
21	CHG 30	16.321	18.014	19.434	20.044	33.763	14.099	20.786	20.015	31.783	24.553
22	CHG 33	15.238	17.090	19.151	17.746	30.183	13.943	21.771	22.071	26.700	24.858
23	DHC 34	23.103	18.367	19.258	17.584	30.718	17.048	18.195	18.228	20.174	21.929
24	DHC 35	22.565	19.002	19.688	17.167	33.965	16.055	20.889	22.323	21.401	22.778
25	DHC 37	20.412	19.155	18.550	17.387	31.888	16.886	19.762	20.840	19.244	22.105
26	DHC 38	24.128	19.254	19.583	17.930	34.898	17.266	19.434	18.681	20.421	22.271
27	DHC 39	22.619	19.997	20.835	16.869	30.624	17.983	20.106	21.816	20.867	22.271
28	ZAP 1	23.198	16.494	21.355	20.253	34.904	18.130	27.172	19.203	16.340	22.058
29	ZAP 2	19.785	16.688	21.265	19.610	36.059	17.718	27.222	18.761	16.059	25.723
30	ZAP 3	20.695	18.157	21.454	20.502	36.064	17.868	27.144	19.211	20.486	26.809
31	ZAP 4	25.664	19.940	19.839	18.813	33.605	16.471	26.166	17.547	19.799	24.798
32	ZAP 5	19.687	16.401	21.097	19.991	34.567	17.589	27.407	18.887	16.074	25.422

### Expression stability analysis of RGs in each livestock species

#### Ladakhi Cattle (LAC)

**Figure 2:**
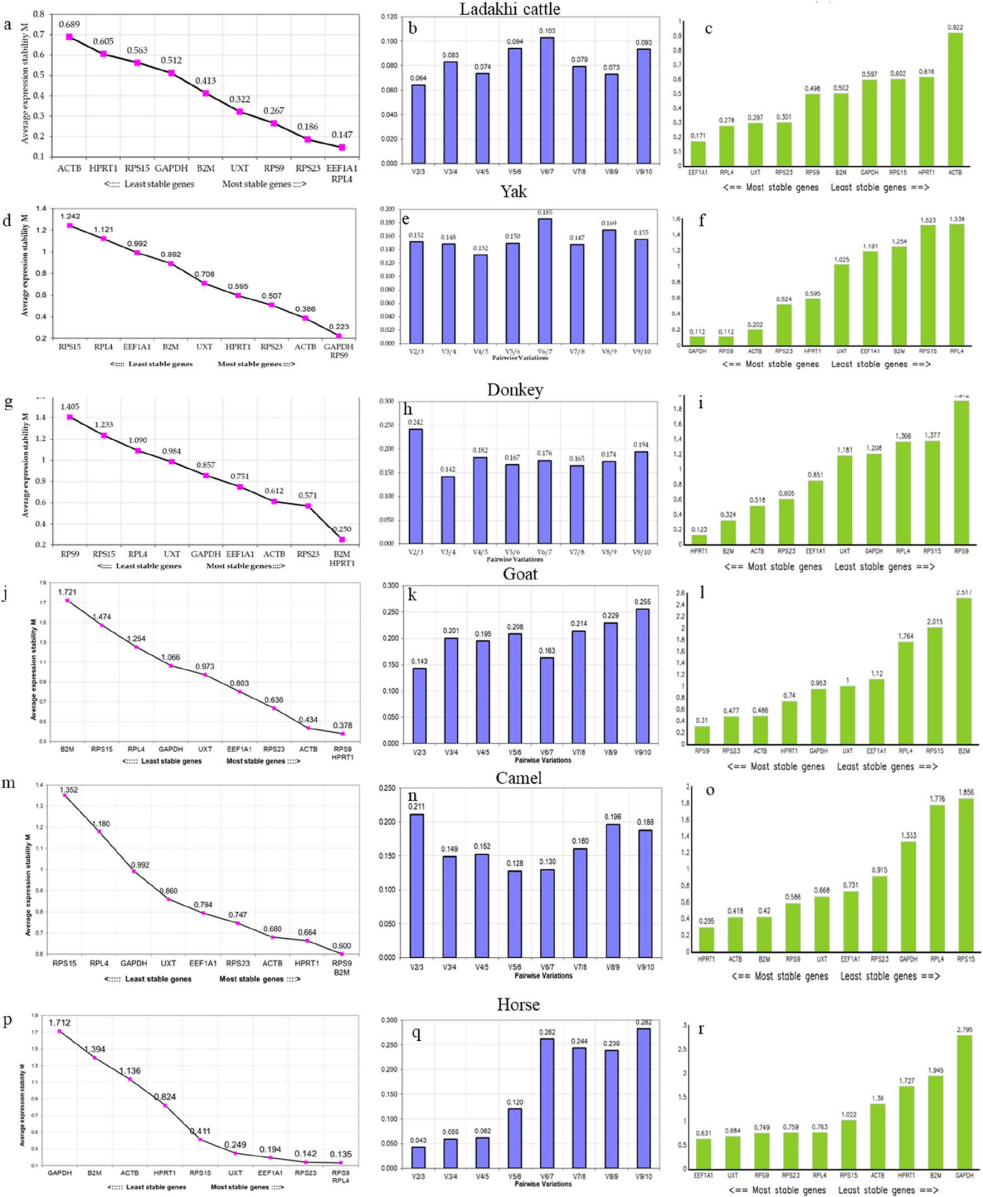
geNorm analysis for ranking of genes based on average expression stability measure (M value), Pair-wise variation (Vn/Vn + 1) between the normalization factors NFn and NFn + 1 to determine the optimal number of reference genes and Normfinder analysis in LAC (**a**–**c**), LAY (**d**–**f**), LAD (**g**–**i**), CHG (**j**–**l**), DHC (**m**–**o**) and ZAP (**p**–**r** respectively).

The geNorm analysis ranked candidate reference genes as per their mean expression stability value (M value) which was below the threshold value of 1.5 for all the 10 RGs*.* The ranking order based on M value were *EEF1A1* = *RPL4* > *RPS23* > *RPS9* > *UXT* > *B2M* > *GAPDH* > *RPS15* > *HPRT1* > *ACTB* (Fig. 2a)*.* The M value ranged from 0.147 (*EEF1A1*) to 0.689 (*ACTB*). The lower M value indicates higher expression stability while higher M value indicates lower expression stability. On the basis of M value, *EEF1A1* = *RPL4* RG pair was most stable expressed while *ACTB* was least stable. Another parameter that was evaluated by geNorm was the pairwise variation Vn/n + 1 in order to calculate the optimal number of RGs to be required for normalization. The pairwise variation (V) score of all the RGs were below 0.15 (Fig. 2b) which is an ideal pairwise recommended score^11^. Therefore, as per V value, combination of two RGs could be suggested to normalize the qPCR data in PBMCs of Ladakhi cattle.

In Normfinder analysis as well, the ranking stability of individual RGs were decided by the lower values indicating higher stability. In LAC, Normfinder analysis resulted in same panel of stable RGs (*EEF1A1, RPL4, UXT, RPS23*) as identified in geNorm analysis. On the other hand, ACTB, HPRT1, RPS15 RGs were identified as least stable. The ranking order from most to least stable RGs was as follows: *EEF1A1* > *RPL4* > *UXT* > *RPS23* > *RPS9* > *B2M* > *GAPDH* > *RPS15* > *HPRT1* > *ACTB* (Fig. 2c, Table 3).

**Table 3:** Overall ranking of best suitable RGs across different species.

Species	Ranking	geNorm	Normfinder	BestKeeper		RefFinder
		M value	Stability value	STDEV	coff. of corr	
Ladakhi cattle (LAC)	1	EEF1A1(0.147)	EEF1A1 (0.171)	RPS15 (0.123)	EEF1A1 (0.978)	EEF1A1 (1.41)
	2	RPL4(0.147)	RPL4 (0.278)	GAPDH (0.163)	RPS9 (0.973)	RPL4 (2.11)
	3	RPS23 (0.186)	UXT (0.297)	B2M (0.391)	RPS23 (0.973)	RPS23 (3.98)
	4	RPS9 (0.267)	RPS23 (0.301)	EEF1A1 (0.468)	RPL4 (0.959)	UXT (4.36)
	5	UXT (0.322)	RPS9 (0.498)	RPL4 (0.481)	UXT (0.941)	RPS15 (4.76)
	6	B2M (0.413)	B2M (0.502)	UXT (0.489)	HPRT1 (0.763)	B2M (5.05)
	7	GAPDH (0.512)	GAPDH (0.597)	RPS23 (0.503)	B2M (0.637)	GAPDH (5.12)
	8	RPS15 (0.563)	RPS15 (0.602)	RPS9 (0.642)	ACTB (0.433)	RPS9 (5.32)
	9	HPRT1 (0.605)	HPRT1 (0.616)	HPRT1 (0.712)	GAPDH (0.017)	HPRT1 (9)
	10	ACTB (0.689)	ACTB (0.922)	ACTB (0.812)	RPS15 (0.001)	ACTB (10)
Ladakhi Yak (LAY)	1	GAPDH (0.223)	GAPDH (0.112)	UXT (0.180)	GAPDH (0.967)	GAPDH (1.41)
	2	RPS9 (0.223)	RPS9 (0.112)	HPRT1 (0.240)	RPS9 (0.965)	RPS9 (2.21)
	3	ACTB (0.386)	ACTB (0.202)	RPL4 (0.500)	EEF1A1 (0.960)	ACTB (3.41)
	4	RPS23 (0.507)	RPS23 (0.524)	GAPDH (0.600)	ACTB (0.929)	HPRT1 (3.76)
	5	HPRT1 (0.595)	HPRT1 (0.595)	ACTB (0.610)	B2M (0.927)	UXT (3.83)
	6	UXT (0.708)	UXT (1.025)	RPS9 (0.680)	RPS23 (0.886)	RPS23 (4.86)
	7	B2M (0.892)	EEF1A1 (1.191)	RPS23 (0.840)	RPS15 (0.813)	RPL4 (7.02)
	8	EEF1A1 (0.992)	B2M (1.254)	B2M (1.350)	HPRT1 (0.727)	EEF1A1 (7.71)
	9	RPL4 (1.121)	RPS15 (1.523)	EEF1A1 (1.590)	UXT (0.001)	B2M (7.74)
	10	RPS15 (1.242)	RPL4 (1.538)	RPS15 (1.750)	RPL4 (0.001)	RPS15 (9.74)
Ladakhi Donkey (LAD)	1	HPRT1(0.250)	HPRT1 (0.123)	ACTB (0.295)	HPRT1 (0.942)	HPRT1 (1.19)
	2	B2M (0.250)	B2M (0.324)	HPRT1 (0.311)	GAPDH (0.941)	B2M (2.00)
	3	RPS23 (0.571)	ACTB (0.518)	RPS23 (0.318)	B2M (0.940)	ACTB (2.45)
	4	ACTB (0.612)	RPS23 (0.605)	B2M (0.472)	EEF1A1 (0.927)	RPS23 (3.46)
	5	EEF1A1 (0.751)	EEF1A1 (0.851)	RPL4 (0.598)	RPS9 (0.619)	EEF1A1 (5.44)
	6	GAPDH (0.857)	UXT (1.181)	UXT (0.613)	RPS15 (0.022)	UXT (6.24)
	7	UXT (0.984)	GAPDH (1.208)	EEF1A1 (0.852)	ACTB (0.001)	RPL4 (7.11)
	8	RPL4 (1.090)	RPL4 (1.366)	RPS15 (0.92)	RPS23 (0.001)	GAPDH (7.17)
	9	RPS15 (1.233)	RPS15 (1.377)	GAPDH (0.95)	UXT (0.00)	RPS15 (8.74)
	10	RPSP (1.405)	RPS9 (1.912)	RPS9 (1.635)	RPL4 (0.001)	RPS9 (10.00)
Chanthangi Goat (CHG)	1	RPS9(0.378)	RPS9 (0.310)	RPS9 (0.1760)	B2M (0.847)	RPS9 (1)
	2	HPRT1 (0.378)	RPS23 (0.477)	HPRT1 (0.324)	RPS23 (0.676)	HPRT1 (2.38)
	3	ACTB (0.434)	ACTB (0.486)	ACTB (0.486)	GAPDH (0.623)	ACTB (2.71)
	4	RPS23 (0.636)	HPRT1 (0.740)	RPS23 (0.595)	RPS15 (0.580)	RPS23 (3.13)
	5	EEF1A1 (0.803)	GAPDH (0.953)	UXT (0.633)	EEF1A1 (0.435)	UXT (5.48)
	6	UXT (0.973)	UXT (1.000)	GAPDH (0.834)	ACTB (0.429)	GAPDH (5.96)
	7	GAPDH (1.066)	EEF1A1 (1.120)	EEF1A1 (0.974)	RPS9 (0.422)	EEF1A1 (6.44)
	8	RPL4 (1.254)	RPL4 (1.764))	RPL4 (1.248)	UXT (0.087)	RPL4 (8)
	9	RPS15 (1.474)	RPS15 (2.015	RPS15 (1.869)	RPL4 (0.051)	RPS15 (9)
	10	B2M (1.721)	B2M (2.517)	B2M (2.301)	HPRT1 (0.001)	B2M (10)
Double hump Camel (DHC)	1	B2M (0.600)	HPRT1 (0.295)	HPRT1 (0.203)	GAPDH (0.372)	HPRT1 (1.32)
	2	RPS9 (0.600)	ACTB (0.418)	EEF1A1 (0.294)	ACTB (0.677)	ACTB (2.63)
	3	HPRT1 (0.664)	B2M (0.420)	ACTB (0.377)	RPS9 (0.751)	B2M (2.71)
	4	ACTB (0.680)	RPS9 (0.586)	RPS23 (0.462)	EEF1A1 (0.001)	RPS9 (2.99)
	5	RPS23 (0.747)	UXT (0.668)	RPS9 (0.542)	RPS15 (0.446)	EEF1A1(4.56)
	6	EEF1A1 (0.794)	EEF1A1 (0.731)	B2M (0.572)	RPS23 (0.055)	RPS23 (5.60)
	7	UXT (0.860)	RPS23 (0.915)	UXT (0.693)	UXT (0.774)	UXT (5.92)
	8	GAPDH (0.992)	RPS15 (1.333)	GAPDH (0.862)	RPL4 (0.599)	GAPDH (8.00)
	9	RPL4 (1.180)	RPL4 (1.766)	RPL4 (1.538)	B2M (0.809)	RPL4 (9.00)
	10	RPS15 (1.352)	RPS15 (1.856)	RPS15 (1.61)	HPRT1 (0.797)	RPS15 (10.00)
Zanskar Horse (ZAP)	1	RPS9(0.135)	EEF1A1 (0.631)	UXT (0.341)	B2M (0.947)	RPS9 (1.73)
	2	RPL4 (0.135)	UXT (0.684)	RPS23 (0.434)	ACTB (0.662)	RPL4 (2.51)
	3	RPS23 (0.142)	RPS9 (0.749)	RPS9 (0.465)	HPRT1 (0.447)	UXT (2.66)
	4	EEF1A1 (0.194)	RPS23 (0.759)	RPL4 (0.469	GAPDH (0.257)	EEF1A1 (2.78)
	5	UXT (0.249)	RPL4 (0.763)	EEF1A1 (0.498)	RPS15 (0.182)	RPS23 (3.13)
	6	RPS15 (0.411)	RPS15 (1.022)	RPS15 (0.816)	EEF1A1 (0.159)	RPS15 (6)
	7	HPRT1 (0.824)	ACTB (1.36)	ACTB (1.211)	RPS9 (0.001)	ACTB (7.24)
	8	ACTB (1.136)	HPRT1 (1.727)	HPRT1 (1.226)	RPS23 (0.001)	HPRT1 (7.74)
	9	B2M (1.394)	B2M (1.945)	B2M (1.914)	UXT (0.001)	B2M (9)
	10	GAPDH (1.712)	GAPDH (2.795)	GAPDH (20.99)	RPL4 (0.001)	GAPDH (10)

The gene expression variation for 10 candidate RGs was also calculated using BestKeeper algorithm. In BestKeeper analysis, raw Ct values were used to evaluate stability of individual RGs based on their SD and CV values. The lower value indicates higher expression stability; however, the SD > 1 value indicates the reference gene is unstable and cannot be used for normalization. The *RPS15* and *GAPDH* genes having lowest SD values of 0.123, 0.163 indicated expression stability. This was followed by *B2M, EEF1A1, RPL4, UXT, RPS23, RPS9, HPRT1* and *ACTB* with SD values 0.391, 0.468, 0.481, 0.491, 0.503, 0.642, 0.712 and 0.812, respectively (Table 4). The *ACTB* gene on the other hand was least stable gene with highest SD value. Additionally, the inter-gene relationship for 10 RGs pairs was also estimated. Strong correlation coefficients (r) were observed for *RPL4/EEF1A1* (0.980), *EEF1A1/RPS9* (0.971), *RPS23/RPS9* (0.966), *RPS23/EEF1A1* (0.962), *RPL4/RPS23* (0.961), *RPL4/ RPS9* (0.922), *UXT/ RPS9* (0.898) (Table 5). This analysis provided strong evidence that these pair of genes have similar expression pattern across the animals. Further, BestKeeper index was calculated for each gene and the correlation between each candidate RGs and BestKeeper was estimated. The relationship between RGs and BestKeeper was described in terms of Pearson correlation coefficient (r), coefficient of determination (r^2^) and the *p* value. The *p* < 0.05 was obtained for all genes indicating a significant contribution of all genes towards the index. Though the *EEF1A1* (0.978) and *RPS9* (0.973) showed high correlation values but their high fold change makes these genes as unreliable reference genes. The statistically significant SD and correlation shown by the RGs from with BestKeeper algorithm appeared to be consistent with their evaluation assessed by geNorm and Normfinder.

**Table 4:** Analysis of parameters based quantitative cycling points (CP) for 10 candidate RGs in LAC.

	*GAPDH*	*ACTB*	*RPS9*	*EEF1A1*	*RPS15*	*RPS23*	*UXT*	*RPL4*	*B2M*	*HPRT1*
n	6	6	6	6	6	6	6	6	6	6
geo Mean [CP]	22.170	23.810	20.460	17.420	19.510	14.410	24.630	20.120	18.550	28.860
AR Mean [CP]	22.170	23.830	20.480	17.440	19.510	14.420	24.640	20.130	18.560	28.870
min [CP]	21.870	22.640	19.870	16.900	19.210	13.940	23.860	19.610	17.740	27.950
max [CP]	22.530	24.900	22.400	18.810	19.790	15.930	26.070	21.560	19.360	30.180
std dev [+/− CP]	0.16	0.81	0.64	0.46	0.12	0.50	0.49	0.48	0.39	0.71
CV [% CP]	0.740	3.410	3.130	2.630	0.630	3.490	1.980	2.390	2.110	2.470
min [x-fold]	− 1.230	− 2.250	− 1.500	− 1.440	− 1.230	− 1.380	− 1.710	− 1.430	− 1.750	− 1.870
max [x-fold]	1.280	2.130	3.840	2.610	1.210	2.880	2.710	2.710	1.750	2.500
std dev [+/− x-fold]	1.120	1.760	1.560	1.370	1.090	1.420	1.400	1.400	1.310	1.640

N = number of samples, geo Mean[CP] = geometric mean of CP; ar Mean[CP] = arithmetic mean of CP; min [CP] and max [CP] = extreme values of CP; Std dev [± CP] = standard deviation of the CP; CV [%CP] = coefficient of variation expressed as a percentage on the C* p* values; min [x-fold] and max [x-fold] = extreme values of expression levels expressed as absolute x-fold over or under coefficient; std dev[± x-fold] = standard deviation of the absolute regulation coefficients.

**Table 5:** Analysis of repeated pair-wise correlation amongst genes in LAC with BestKeeper index.

	*GAPDH*	*ACTB*	*RPS9*	*EEF1A1*	*RPS15*	*RPS23*	*UXT*	*RPL4*	*B2M*	*HPRT1*
*ACTB*	− 0.107	–	–	–	–	–	–	–	–	–
*p *value	0.84	–	–	–	–	–	–	–	–	–
*RPS9*	− 0.08	0.309	–	–	–	–	–	–	–	–
*p *value	0.88	0.552	–	–	–	–	–	–	–	–
*EEF1A1*	− 0.056	0.316	0.971	–	–	–	–	–	–	–
*p *value	0.916	0.542	0.001	–	–	–	–	–	–	–
*RPS15*	− 0.856	0.072	− 0.014	0.08	–	–	–	–	–	–
*p *value	0.03	0.892	0.979	0.881	–	–	–	–	–	–
*RPS23*	− 0.14	0.252	0.966	0.962	0.137	–	–	–	–	–
*p *value	0.791	0.631	0.002	0.002	0.796	–	–	–	–	–
*UXT*	0.339	0.378	0.898	0.888	− 0.344	0.849	–	–	–	–
*p *value	0.511	0.46	0.015	0.018	0.504	0.033	–	–	–	–
*RPL4*	− 0.102	0.34	0.922	0.98	0.206	0.961	0.84	–	–	–
*p *value	0.848	0.51	0.009	0.001	0.695	0.002	0.036	–	–	–
*B2M*	0.396	− 0.148	0.585	0.611	− 0.197	0.698	0.702	0.652	–	–
*p *value	0.436	0.779	0.222	0.197	0.709	0.123	0.12	0.16	–	–
*HPRT1*	0.052	0.4	0.82	0.747	− 0.317	0.645	0.775	0.612	0.162	–
*p *value	0.922	0.432	0.046	0.088	0.541	0.166	0.07	0.197	0.759	–

BestKeeper vs	*GAPDH*	*ACTB*	*RPS9*	*EEF1A1*	*RPS15*	*RPS23*	*UXT*	*RPL4*	*B2M*	*HPRT1*
coeff. of corr. [r]	0.017	0.433	0.973	0.978	0.001	0.959	0.941	0.959	0.637	0.763
*p *value	0.975	0.392	0.001	0.001	0.971	0.003	0.005	0.003	0.173	0.078

Additionally, RefFinder based analysis was carried out that ranks the stability order of RGs in a more refined way by taking into consideration geNorm, Normfinder, BestKeeper and delta Ct algorithms. The stability order and ranking of the RGs as per RefFinder were; *EEF1A1* (1.41) *RPL4* (2.11), *RPS23* (3.98), *UXT* (4.36), *RPS15* (4.76), *B2M* (5.05), *GAPDH* (5.12), *RPS9* (5.32), *HPRT1* (9), *ACTB* (10).

#### Ladakhi Yak (LAY)

The M value for all the 10 genes in geNorm analysis were found to be within acceptable range in LAY. The ranking order of RGs was *GAPDH* = *RPS9* > *ACTB* > *RPS23* > *HPRT1* > *UXT* > *B2M* > *EEF1A1* > *RPL4* > *RPS15* (Fig. 2d)*. GAPDH* and *RPS9* showed higher gene expression stability with M value of 0.223 followed by *ACTB, RPS23* and *HPRT1* with M value of 0.386, 0.507, 0.595 respectively (Table 3). On the other hand, *RPS15*, *RPL4* and *EEF1A1* were least stable with higher M values of 1.242, 1.121 and 0.992, respectively. The pair wise variation analysis showed V4/5 combination with least V value (0.132) followed by V3/4 (0.148) and V5/V6 (0.150) combinations (Fig. 2e). Since all these V values were well within the acceptable range (recommended cut-off value 0.15), therefore use of panel of 3 RGs *(GAPDH, RPS9* and *ACTB*) is likely to provide most accurate normalization in Ladakhi yak samples. The Normfinder analysis also identified same set of RGs in LAY samples with highest stability; *GAPDH* (0.112), *RPS9* (0.112) and *ACTB* (0.202) albeit slight change in their ranking order; *GAPDH* > *RPS9* > *ACTB* > *RPS23* > *HPRT1* > *UXT* > *EEF1A1* > *B2M* > *RPS15* > *RPL4* (Fig. 2f, Table 3). Similar to geNorm, *RPL4* (1.538) and *RPS15* (1.523) were found to be least stable RGs.

In BestKeeper analysis, *UXT* was found to be most stable with minimum SD value (0.180) followed by *HPRT1, RPL4, GAPDH, ACTB, RPS9, RPS23, B2M, EEF1A1, RPS15* with the SD values of 0.240, 0.500, 0.600, 0.610, 0.680, 0.840, 1.350, 1.590, 1.750, respectively (Table 6). Additionally, high correlation coefficient was observed for *RPS9/GAPDH* (r = 0.973), *B2M/ACTB* (r = 0.942), *EEF1A1/GAPDH* (r = 0.931), *EEF1A1/RPS9* (r = 0.923), *RPS23/ACTB* (r = 0.914) and *B2M/EEF1A1* (r = 0.909) pair combinations. The best correlation between RGs and BestKeeper was observed for *GAPDH* (r = 0.967), *RPS9* (r = 0.965), *EEF1A1* (r = 0.960), *ACTB* (r = 0.929), *B2M* (r = 0.927) (Table 7). The high correlation values for these genes indicated their reliability as RGs, The *GAPDH, RPS9* and *ACTB* were termed as best RGs on the basis of highest correlation value and less SD.

**Table 6:** Analysis of parameters based quantitative cycling points (CP) for 10 candidate RGs in LAY.

	*GAPDH*	*ACTB*	*RPS9*	*EEF1A1*	*RPS15*	*RPS23*	*UXT*	*RPL4*	*B2M*	*HPRT1*
n	6	6	6	6	6	6	6	6	6	6
geo Mean [CP]	18.29	20.97	22.18	22.16	31.87	16.13	22.76	24.31	21.44	24.92
AR Mean [CP]	18.31	20.98	22.2	22.23	31.93	16.16	22.76	24.32	21.51	24.93
min [CP]	17.34	19.62	21.06	19.57	29.06	14.37	22.22	23.71	18.11	24.33
max [CP]	19.2	22.12	23.42	23.62	33.82	17.51	23.04	25.65	22.95	25.41
std dev [+/− CP]	0.60	0.61	0.68	1.59	1.75	0.84	0.18	0.50	1.35	0.24
CV [% CP]	3.3	2.9	3.06	7.14	5.47	5.21	0.8	2.06	6.29	0.98
min [x-fold]	− 1.94	− 2.54	− 2.18	− 6.02	− 7.04	− 3.39	− 1.46	− 1.52	− 10.08	− 1.51
max [x-fold]	1.88	2.22	2.36	2.75	3.85	2.6	1.21	2.53	2.84	1.4
std dev [+/− x-fold]	1.52	1.52	1.6	3.01	3.35	1.79	1.13	1.42	2.55	1.18

**Table 7:** Analysis of repeated pair-wise correlation amongst genes in LAY with BestKeeper index.

	*GAPDH*	*ACTB*	*RPS9*	*EEF1A1*	*RPS15*	*RPS23*	*UXT*	*RPL4*	*B2M*	*HPRT1*
*ACTB*	0.859	–	–	–	–	–	–	–	–	–
*p *value	0.028	–	–	–	–	–	–	–	–	–
*RPS9*	0.973	0.84	–	–	–	–	–	–	–	–
*p *value	0.001	0.037	–	–	–	–	–	–	–	–
*EEF1A1*	0.931	0.887	0.923	–	–	–	–	–	–	–
*p *value	0.007	0.019	0.009	–	–	–	–	–	–	–
*RPS15*	0.892	0.632	0.805	0.751	–	–	–	–	–	–
*p *value	0.017	0.178	0.053	0.085	–	–	–	–	–	–
*RPS23*	0.784	0.914	0.755	0.786	0.693	–	–	–	–	–
*p *value	0.065	0.011	0.083	0.064	0.127	–	–	–	–	–
*UXT*	− 0.437	− 0.581	− 0.265	− 0.31	− 0.525	− 0.652	–	–	–	–
*p *value	0.386	0.227	0.611	0.55	0.285	0.161	–	–	–	–
*RPL4*	− 0.565	− 0.792	− 0.597	− 0.799	− 0.322	− 0.761	0.207	–	–	–
*p *value	0.243	0.06	0.211	0.056	0.533	0.079	0.694	–	–	–
*B2M*	0.82	0.942	0.87	0.909	0.538	0.858	− 0.292	− 0.879	–	–
*p *value	0.046	0.005	0.024	0.012	0.271	0.029	0.574	0.021	–	–
*HPRT1*	0.62	0.602	0.777	0.671	0.371	0.587	0.186	− 0.634	0.81	–
*p *value	0.19	0.206	0.069	0.145	0.47	0.221	0.724	0.176	0.051	–

BestKeeper vs	*GAPDH*	*ACTB*	*RPS9*	*EEF1A1*	*RPS15*	*RPS23*	*UXT*	*RPL4*	*B2M*	*HPRT1*
coeff. of corr. [r]	0.967	0.929	0.965	0.96	0.813	0.886	0.001	0.001	0.927	0.727
*p *value	0.002	0.007	0.002	0.002	0.049	0.019	0.401	0.096	0.008	0.102

The RefFinder analysis also identified *GAPDH*, *RPS9* and *ACTB* to be most stable RGs while *RPS15*, *B2M* and *EEF1A1* were the least stable RGS in LAY. In the present investigation, all four methods geNorm, Normfinder BestKeeper and RefFinder have demonstrated that *GAPDH*, *RPS9* and *ACTB* are the most stable RGs in PBMCs of LAY.

#### Ladakhi Donkey (LAD)

In Ladakhi donkey as well, the geNorm analysis showed mean expression stability values of 10 RGs within the acceptable range and varied from 0.250 (*HPRT1* = *B2M*) to 1.405 (*RPS9*) (Table 3). The stability ranking of RGs was: *HPRT1* = *B2M* > *RPS23* > *ACTB* > *EEF1A1* > *GAPDH* > *UXT* > *RPL4* > *RPS15* > *RPS9* (Fig. 2 g). The *B2M* and *HPRT1* RGs showed highest expression stability with lowest M value while *RPS9* and *RPS15* RGs showed least expression stability with highest M value. Based on pair-wise variation analysis (V value), V3/4 combination (*B2M HPRT1* and *RPS23*) with V value of 0.142 was found to provide the most accurate normalization in Ladakhi donkey (Fig. 2 h). In Normfinder analysis as well; *HPRT1* (0.123), *B2M* (0.324) and *ACTB* (0.518) were most stable with lowest values (Fig. 2i). On the other hand, the *RPS9* (1.912), *RPS15* (1.377) and *RPL4* (1.366) RGs were least stable.

The BestKeeper analysis showed *ACTB* gene to be most stable with the lowest crossing point SD value of 0.295. This was followed by *HPRT1, RPS23* and *B2M* RGs with SD value of 0.311, 0.318 and 0.472, respectively. On the other hand, *RPS9* with highest crossing point SD value of 1.635 was found to be the least stable (Table 8). In addition, the inter-gene relation for 10 RGs pairs was also estimated. *B2M/GAPDH* (r = 1.0), *HPRT1/B2M* (r = 0.985), *HPRT1/GAPDH* (r = 0.985), *B2M/EEF1A1* (r = 0.855) and *EEF1A1/GAPDH* (r = 0.854) showed the strong correlation coefficients (Table 9). The highly correlated RGs were combined into BestKeeper index and the correlation between each candidate RGs and BestKeeper was estimated. The relationship between RG and BestKeeper was described in terms of Pearson correlation coefficient (r), coefficient of determination correlation between BestKeeper and RGs was observed for *HPRT1* (r = 0.942) and *GAPDH* (r = 0.941) followed by *B2M* (0.940) and *EEF1A1* (0.927) genes. The statistically significant correlation shown by RGs (*HPRT1, B2M)* with the BestKeeper index appeared to be consistent with their evaluation as assessed by geNorm and Normfinder. RefFinder was another tool, were evaluating and identified RGs from comprehensive data set. *HPRT1*, *B2M* and *ACTB* were most stable and *RPS9*, *RPS15* and *GAPDH* were least stable genes identified by RefFinder in LAD.

**Table 8:** Analysis of parameters based quantitative cycling points (CP) for 10 candidate RGs in LAD.

	*GAPDH*	*ACTB*	*RPS9*	*EEF1A1*	*RPS15*	*RPS23*	*UXT*	*RPL4*	*B2M*	*HPRT1*
n	5	5	5	5	5	5	5	5	5	5
geo Mean [CP]	22.83	20.66	21.35	19.45	33.76	14.33	22.69	21.66	17.74	25.56
AR Mean [CP]	22.87	20.66	21.43	19.48	33.78	14.34	22.71	21.68	17.75	25.57
min [CP]	20.73	19.97	19.7	17.94	31.86	13.86	21.45	20.55	16.69	24.79
max [CP]	24.53	21.09	24.16	20.65	35.47	14.84	23.92	23.17	18.58	26.04
std dev [+/− CP]	0.95	0.30	1.64	0.85	0.92	0.32	0.61	0.60	0.47	0.31
CV [% CP]	4.16	1.43	7.63	4.37	2.72	2.22	2.7	2.76	2.66	1.22
min [x-fold]	− 4.3	− 1.61	− 3.15	− 2.86	− 3.73	− 1.39	− 2.36	− 2.16	− 2.07	− 1.71
max [x-fold]	3.24	1.35	6.99	2.29	3.27	1.42	2.34	2.85	1.79	1.39
std dev [+/− x-fold]	1.93	1.23	3.11	1.81	1.89	1.25	1.53	1.51	1.39	1.24

**Table 9:** Analysis of repeated pair-wise correlation amongst genes in LAD with BestKeeper index.

	*GAPDH*	*ACTB*	*RPS9*	*EEF1A1*	*RPS15*	*RPS23*	*UXT*	*RPL4*	*B2M*	*HPRT1*
*ACTB*	− 0.067	–	–	–	–	–	–	–	–	–
*p *value	0.915	–	–	–	–	–	–	–	–	–
*RPS9*	0.345	− 0.434	–	–	–	–	–	–	–	–
*p *value	0.57	0.465	–	–	–	–	–	–	–	–
*EEF1A1*	0.854	0.096	0.608	–	–	–	–	–	–	–
*p *value	0.065	0.878	0.276	–	–	–	–	–	–	–
*RPS15*	− 0.301	− 0.134	0.629	− 0.036	–	–	–	–	–	–
*p *value	0.623	0.83	0.256	0.954	–	–	–	–	–	–
*RPS23*	− 0.004	0.363	− 0.793	− 0.171	− 0.931	–	–	–	–	–
*p *value	0.995	0.548	0.109	0.783	0.022	–	–	–	–	–
*UXT*	− 0.281	0.204	− 0.827	− 0.674	− 0.289	0.372	–	–	–	–
*p *value	0.647	0.742	0.084	0.212	0.638	0.538	–	–	–	–
*RPL4*	− 0.71	− 0.162	− 0.709	− 0.895	− 0.353	0.51	0.581	–	–	–
*p *value	0.179	0.795	0.18	0.04	0.56	0.381	0.304	–	–	–
*B2M*	1	− 0.065	0.343	0.855	− 0.304	0	− 0.282	− 0.709	–	–
*p *value	0.001	0.918	0.572	0.065	0.619	1	0.646	0.18	–	–
*HPRT1*	0.985	− 0.04	0.339	0.814	− 0.206	− 0.094	− 0.191	− 0.739	0.985	–
*p *value	0.002	0.949	0.577	0.094	0.74	0.881	0.758	0.153	0.002	–

BestKeeper vs	*GAPDH*	*ACTB*	*RPS9*	*EEF1A1*	*RPS15*	*RPS23*	*UXT*	*RPL4*	*B2M*	*HPRT1*
coeff. of corr. [r]	0.941	0.001	0.619	0.927	0.022	0.001	0.001	0.001	0.94	0.942
*p *value	0.017	0.863	0.265	0.024	0.972	0.612	0.406	0.046	0.018	0.017

#### Chanthangi Goat (CHG)

The geNorm analysis of all the 10 candidate RGs in Changthangi goat exhibited mean expression stability (M) values well below 1.5 (Table 3). The stability ranking RGs were in the following order; *RPS9* = *HPRT* > *ACTB* > *RPS23* > *EEF1A1* > *UXT* > *GAPDH* > *RPL4* > *RPS15* > *B2M* (Fig. 2j). The *RPS9* and *HPRT* were most stable with lowest M value of 0.378 while *RPS15* and *B2M* had maximum expression variability and highest M values of 1.474 and 1.721 respectively.

Further, the pair-wise variation analysis provided within the acceptable limit on sequential addition of another gene to the two most stably expressed genes, viz., *B2M* and *HPRT1* the pair-wise combination V2/3 gave the acceptable V value of 0.143 (< 0.15) suggesting that the geometric mean between *RPS9*, *HPRT1* and *ACTB* is optimal for data normalization in Changthangi goat (Fig. 2k). Similar to geNorm, Normfinder also identified *RPS9* (0.310)*, RPS23* (0.477)*, ACTB* (0.486) and *HPRT1* (0.740) as most stable and *B2M* (2.517) and *RPS15* (2.015) as least stably expressed genes (Fig. 2l, Table 3). There was good agreement between geNorm and Normfinder outcome, albeit slight variation was observed in the ranking of RGs. The BestKeeper algorithm showed consistent expression levels for all the RGs. *RPS9* (0.176)*,* exhibited low SD and 0.422 correlation coefficients in BestKeeper analysis, pointing towards their expression stability (Table 10). Additionally, *RPS9/ACTB* (r = 0.974), *B2M/RPS23* (r = 0.801), *B2M/EEF1A1* (r = 0.739), and *RPS23/GAPDH* (r = 0.712) showed the strong correlation coefficients (Table 11). *B2M* (0.847) showed the high correlation value but they showed the high fold change thus their reliability as a RGs is not applicable. RefFinder were identified the overall ranking of the gene. The ranking of genes was *RPS9* (1), *HPRT1* (2.38), *ACTB* (2.71), *RPS23* (3.13), *UXT* (5.48), *GAPDH* (5.96), *EEF1A1* (6.44), *RPL4* (8), *RPS15* (9), *B2M* (10). In the present investigation, all four algorithmic methods geNorm Normfinder, BestKeeper and RefFinder have demonstrated that *RPS9*, *HPRT1* and *ACTB* are the most stable RGs in CHG.

**Table 10:** Analysis of parameters based quantitative cycling points (CP) for 10 candidate RGs in CHG.

	*GAPDH*	*ACTB*	*RPS9*	*EEF1A1*	*RPS15*	*RPS23*	*UXT*	*RPL4*	*B2M*	*HPRT1*
n	5	5	5	5	5	5	5	5	5	5
geo Mean [CP]	15.8	17.92	19.46	19.09	32.82	14.37	21.39	21.92	30.29	24.86
AR Mean [CP]	15.84	17.93	19.46	19.12	32.88	14.39	21.41	21.97	30.39	24.86
min [CP]	14.35	17.09	19.15	17.75	30.18	13.63	20.44	20.01	26.7	24.36
max [CP]	17.4	18.64	19.79	20.64	34.92	15.23	22.62	23.87	33.05	25.45
std dev [+ /− CP]	0.83	0.49	0.18	0.97	1.87	0.6	0.63	1.25	2.3	0.32
CV [% CP]	5.27	2.71	0.9	5.1	5.68	4.14	2.96	5.68	7.57	1.3
min [x-fold]	− 2.74	− 1.78	− 1.24	− 2.54	− 6.22	− 1.67	− 1.94	− 3.77	− 12	− 1.41
max [x-fold]	3.02	1.64	1.26	2.92	4.3	1.81	2.34	3.85	6.8	1.51
std dev [+ /− x-fold]	1.78	1.4	1.13	1.96	3.65	1.51	1.55	2.38	4.93	1.25

**Table 11:** Analysis of repeated pair-wise correlation amongst genes in CHG with BestKeeper index.

	*GAPDH*	*ACTB*	*RPS9*	*EEF1A1*	*RPS15*	*RPS23*	*UXT*	*RPL4*	*B2M*	*HPRT1*
*ACTB*	− 0.379	–	–	–	–	–	–	–	–	–
*p *value	0.53	–	–	–	–	–	–	–	–	–
*RPS9*	− 0.431	0.974	–	–	–	–	–	–	–	–
*p *value	0.468	0.005	–	–	–	–	–	–	–	–
*EEF1A1*	0.096	0.663	0.482	–	–	–	–	–	–	–
*p *value	0.878	0.222	0.412	–	–	–	–	–	–	–
*RPS15*	0.163	0.422	0.522	− 0.041	–	–	–	–	–	–
*p *value	0.793	0.479	0.367	0.948	–	–	–	–	–	–
*RPS23*	0.712	− 0.035	− 0.11	0.398	− 0.185	–	–	–	–	–
*p *value	0.177	0.956	0.86	0.507	0.766	–	–	–	–	–
*UXT*	0.341	− 0.574	− 0.393	− 0.852	0.335	− 0.009	–	–	–	–
*p *value	0.574	0.311	0.513	0.067	0.581	0.988	–	–	–	–
*RPL4*	− 0.293	0.05	0.274	− 0.673	0.441	− 0.264	0.689	–	–	–
*p *value	0.632	0.937	0.655	0.213	0.457	0.668	0.199	–	–	–
*B2M*	0.697	0.334	0.208	0.739	0.194	0.801	− 0.292	− 0.475	–	–
*p *value	0.191	0.583	0.737	0.154	0.754	0.103	0.633	0.419	–	–
*HPRT1*	− 0.625	0.533	0.478	0.456	− 0.494	0.081	− 0.669	− 0.08	− 0.034	–
*p *value	0.26	0.355	0.415	0.44	0.398	0.897	0.217	0.898	0.956	–

BestKeeper vs	*GAPDH*	*ACTB*	*RPS9*	*EEF1A1*	*RPS15*	*RPS23*	*UXT*	*RPL4*	*B2M*	*HPRT1*
coeff. of corr. [r]	0.623	0.429	0.422	0.435	0.58	0.676	0.087	0.051	0.847	0.001
*p *value	0.262	0.471	0.479	0.464	0.305	0.211	0.89	0.935	0.07	0.783

#### Double hump Camel (DHC)

The geNorm analysis of 10 RGs showed M values ranging from 0.600 to 1.352 in double hump camel (Table 3). The M values for all the RGs were within the acceptable limit of < 1.5. On the basis of relative expression stability and stepwise exclusion, the ranking order of RGs was: *B2M* = *RPS9* > *HPRT1* > *ACTB* > *RPS23* > *EEF1A1* > *UXT* > *GAPDH* > *RPL4* > *RPS15* (Fig. 2m). The expression of *RPS9* and *B2M* RGs with lowest M values of 0.600 were found to be most stable while *RPL4* and *RPS15* RGs with highest M values of 1.180 and 1.352, respectively were found to be least stable RGs in DHC. Based on pair-wise combination, the V values for V3/4, V5/6 and V6/7 and were close to the threshold value of 0.15. Therefore, the combination of V3/4 with *ACTB*, *HPRT1* and *B2M* RGs should provide the accurate normalization of qPCR data in DHC (Fig. 2n).

In Normfinder analysis, the RGs were ranked as follows: *HPRT1* > *ACTB* > *B2M* > *RPS9* > *UXT* > *EEF1A1* > *RPS23* > *GAPDH* > *RPL4* > *RPS15* (Fig. 2o). The *HPRT1* (0.295), *ACTB* (0.418*), B2M* (0.420), *RPS9* (0.586) were four most stable RGs as per stability values.

In BestKeeper analysis, *HPRT1* gene with the lowest crossing point SD value of 0.203 was found to be most stable. This was followed by *EEF1A1, ACTB* and *RPS23* genes with SD values of 0.294, 0.377, and 0.462, respectively (Table12). On the other hand, *RPS15, RPL4* and *GAPDH* RGs with high crossing point SD values of 1.61, 1.54, 0.86 respectively were found to be least stable. Strong correlation was observed in inter gene relationship of the RGs *RPL4/UXT* (r = 0.908), *HPRT1/UXT* (r = 0.884) and *HPRT1/B2M* (r = 0.755) (Table 13). The relationship between RGs and BestKeeper was described in terms of Pearson correlation coefficient (r), coefficient of determination correlation between BestKeeper and RGs was observed for *HPRT1* (r = 0.797) and *B2M* (r = 0.809) followed by *UXT*, *RPS9* and *ACTB* gene.

**Table 12:** Analysis of parameters based quantitative cycling points (CP) for 10 candidate RGs in DHC.

	*GAPDH*	*ACTB*	*RPS9*	*EEF1A1*	*RPS15*	*RPS23*	*UXT*	*RPL4*	*B2M*	*HPRT1*
n	5	5	5	5	5	5	5	5	5	5
geo Mean [CP]	22.53	19.15	19.57	17.38	32.37	17.04	19.66	20.31	20.41	22.27
AR Mean [CP]	22.57	19.16	19.58	17.39	32.42	17.05	19.68	20.38	20.42	22.27
min [CP]	20.41	18.37	18.55	16.87	30.62	16.05	18.19	18.23	19.24	21.93
max [CP]	24.13	20	20.83	17.93	34.9	17.98	20.89	22.32	21.4	22.78
std dev [+/− CP]	0.86	0.38	0.54	0.29	1.61	0.46	0.69	1.54	0.57	0.2
CV [% CP]	3.82	1.97	2.77	1.69	4.97	2.71	3.52	7.55	2.8	0.91
min [x-fold]	− 4.35	− 1.72	− 2.03	− 1.43	− 3.37	− 1.98	− 2.76	− 4.23	− 2.25	− 1.27
max [x-fold]	3.03	1.8	2.4	1.46	5.77	1.92	2.35	4.03	1.99	1.42
std dev [+ /− x-fold]	1.82	1.3	1.46	1.23	3.05	1.38	1.62	2.9	1.49	1.15

**Table 13:** Analysis of repeated pair-wise correlation amongst genes in DHC with BestKeeper index.

	*GAPDH*	*ACTB*	*RPS9*	*EEF1A1*	*RPS15*	*RPS23*	*UXT*	*RPL4*	*B2M*	*HPRT1*
*ACTB*	− 0.075	–	–	–	–	–	–	–	–	–
*p *value	0.905	–	–	–	–	–	–	–	–	–
*RPS9*	0.471	0.665	–	–	–	–	–	–	–	–
*p *value	0.423	0.22	–	–	–	–	–	–	–	–
*EEF1A1*	0.42	− 0.536	− 0.552	–	–	–	–	–	–	–
*p *value	0.482	0.352	0.334	–	–	–	–	–	–	–
*RPS15*	0.383	− 0.043	− 0.155	0.519	–	–	–	–	–	–
*p *value	0.524	0.946	0.804	0.37	–	–	–	–	–	–
*RPS23*	0.194	0.591	0.528	− 0.129	− 0.48	–	–	–	–	–
*p *value	0.754	0.294	0.36	0.836	0.413	–	–	–	–	–
*UXT*	− 0.247	0.571	0.324	− 0.569	0.386	− 0.318	–	–	–	–
*p *value	0.689	0.315	0.594	0.317	0.521	0.602	–	–	–	–
*RPL4*	− 0.471	0.569	0.366	− 0.845	− 0.029	− 0.204	0.908	–	–	–
*p *value	0.423	0.317	0.545	0.071	0.963	0.741	0.033	–	–	–
*B2M*	0.555	0.222	0.734	− 0.382	0.28	− 0.165	0.516	0.427	–	–
*p *value	0.332	0.72	0.158	0.526	0.648	0.79	0.373	0.473	–	–
*HPRT1*	0.096	0.253	0.315	− 0.347	0.592	− 0.549	0.884	0.706	0.755	–
*p *value	0.878	0.681	0.606	0.567	0.293	0.338	0.047	0.183	0.14	–

BestKeeper vs	*GAPDH*	*ACTB*	*RPS9*	*EEF1A1*	*RPS15*	*RPS23*	*UXT*	*RPL4*	*B2M*	*HPRT1*
coeff. of corr. [r]	0.372	0.677	0.751	0.001	0.446	0.055	0.774	0.599	0.809	0.797
*p *value	0.538	0.21	0.144	0.51	0.451	0.93	0.124	0.285	0.097	0.107

RefFinder based overall analysis resulted in stability ranking of RGs as; *HPRT1* (1.32) > *ACTB* (2.63) >  *B2M* (2.71) >  *RPS9* (2.99) > *EEF1A1* (4.56) > *RPS23* (5.60) > *UXT* (5.92) > *GAPDH* (8.00) > *RPL4* (9.00) > *RPS15* (10.00). Overall, *HPRT1, B2M* and *ACTB* were identified as the most appropriate RGs in high altitude adapted DHC using all four algorithms.

#### Zanskar Horses (ZAP)

The M values calculated using geNorm analysis for all the RGs in Zanskar ponies are shown in Table 3. Except, *B2M* and *GAPDH* RGs, the M values for all other RGs were within the acceptable limit of < 1.5. The M value for all the RGs in ZAP ranged from 0.135 to 1.721. The ranking order of RGs was as follows; *RPS9* = *RPL4* > *RPS23* > *EEF1A1* > *UXT* > *RPS15* > *HPRT1* > *ACTB* > *B2M* > *GAPDH* (Fig. 2p). The two most stable RGs with lowest M value were *RPS9* and *RPL4* (0.135) while *GAPDH* and *B2M* were the least stable RGs with M value of 1.712 and 1.394 respectively. Further, the V values for V2/3, V3/4, V4/5 and V5/6 were within the threshold limit of 0.15. Based on geNorm analysis, the geometric mean of *RPS9, RPL4* and *RPS23* RGs is likely to provide accurate normalization of gene expression data in ZAP (Fig. 2q).

In Normfinder analysis ranking of genes in high altitude ZAP from most stable to least stable was as follows: *EEF1A1* (0.631), *UXT* (0.684), *RPS9* (0.749), *RPS23* (0.759), *RPL4* (0.763), *RPS15* (1.022), *ACTB* (1.36), *HPRT1* (1.727), *B2M* (1.945), *GAPDH* (2.795) (Fig. 2r).

From BestKeeper algorthim, *UXT* gene revealed minimum SD value of 0.341 with smallest variation, followed by *RPS23, RPS9, RPL4, EEF1A1, RPS15, ACTB, HPRT1, B2M* and *GAPDH* with the SD value 0.434, 0.465, 0.469, 0.498, 0.816, 1.211, 1.226, 1.914, 2.099 respectively (Table 14). The best correlation between RGs and BestKeeper was observed for *B2M* (r = 0.947) and *ACTB* (r = 0.662) (Table 15). The high correlation values for these genes indicated their reliability as RGs.

**Table 14:** Analysis of parameters based quantitative cycling points (CP) for 10 candidate RGs in ZAP.

	*GAPDH*	*ACTB*	*RPS9*	*EEF1A1*	*RPS15*	*RPS23*	*UXT*	*RPL4*	*B2M*	*HPRT1*
n	5	5	5	5	5	5	5	5	5	5
geo Mean [CP]	21.69	17.49	20.99	19.82	35.03	17.55	27.02	18.71	17.65	24.91
AR Mean [CP]	21.81	17.54	21	19.83	35.04	17.56	27.02	18.72	17.75	24.96
min [CP]	19.69	16.4	19.84	18.81	33.61	16.47	26.17	17.55	16.06	22.06
max [CP]	25.66	19.94	21.45	20.5	36.06	18.13	27.41	19.21	20.49	26.81
std dev [+/− CP]	2.1	1.21	0.46	0.5	0.82	0.43	0.34	0.47	1.91	1.23
CV [% CP]	9.63	6.91	2.21	2.51	2.33	2.47	1.26	2.5	10.78	4.91
min [x-fold]	− 4	− 2.12	− 2.22	− 2.02	− 2.67	− 2.11	− 1.8	− 2.24	− 3	− 7.21
max [x-fold]	15.68	5.48	1.37	1.6	2.05	1.5	1.31	1.41	7.18	3.73
std dev [+ /− x-fold]	4.28	2.32	1.38	1.41	1.76	1.35	1.27	1.38	3.77	2.34

**Table 15:** Analysis of repeated pair-wise correlation amongst genes in ZAP with BestKeeper index.

	*GAPDH*	*ACTB*	*RPS9*	*EEF1A1*	*RPS15*	*RPS23*	*UXT*	*RPL4*	*B2M*	*HPRT1*
*ACTB*	0.715	–	–	–	–	–	–	–	–	–
*p *value	0.175	–	–	–	–	–	–	–	–	–
*RPS9*	− 0.762	− 0.798	–	–	–	–	–	–	–	–
*p *value	0.134	0.106	–	–	–	–	–	–	–	–
*EEF1A1*	− 0.586	− 0.615	0.913	–	–	–	–	–	–	–
*p *value	0.299	0.27	0.031	–	–	–	–	–	–	–
*RPS15*	− 0.734	− 0.469	0.826	0.647	–	–	–	–	–	–
*p *value	0.158	0.425	0.085	0.238	–	–	–	–	–	–
*RPS23*	− 0.628	− 0.82	0.976	0.906	0.728	–	–	–	–	–
*p *value	0.257	0.089	0.004	0.034	0.164	–	–	–	–	–
*UXT*	− 0.881	− 0.922	0.917	0.8	0.656	0.876	–	–	–	–
*p *value	0.049	0.026	0.028	0.104	0.229	0.051	–	–	–	–
*RPL4*	− 0.685	− 0.777	0.981	0.966	0.723	0.979	0.899	–	–	–
*p *value	0.202	0.122	0.003	0.008	0.167	0.004	0.038	–	–	–
*B2M*	0.434	0.854	− 0.388	− 0.128	− 0.095	− 0.446	− 0.61	− 0.347	–	–
*p *value	0.466	0.065	0.519	0.837	0.879	0.452	0.275	0.568	–	–
*HPRT1*	− 0.503	0.244	0.046	0.019	0.415	− 0.169	0.073	− 0.039	0.433	–
*p *value	0.387	0.693	0.941	0.975	0.488	0.786	0.907	0.95	0.467	–

BestKeeper vs	*GAPDH*	*ACTB*	*RPS9*	*EEF1A1*	*RPS15*	*RPS23*	*UXT*	*RPL4*	*B2M*	*HPRT1*
coeff. of corr. [r]	0.257	0.662	0.001	0.159	0.182	0.001	0.001	0.001	0.947	0.427
*p *value	0.676	0.224	0.894	0.799	0.77	0.821	0.533	0.939	0.014	0.473

In RefFinder analysis, *RPS9*, *RPL4* and *UXT* were overall most stable while *GAPDH*, *B2M* and *HPRT1* were the least stable. Based on all the methods; geNorm Normfinder, BestKeeper and RefFinder *RPS9, RPL4* and UXT were observed to be most stable RGs in ZAP.

## Discussion

These days, identification of appropriate RGs is a fundamental part of gene expression studies. It has been suggested in many reports^10,26,38^ that there are no panel of RGs that can be used universally for normalization of gene expression data. Several studies have been highlighted the importance of proper RGs for normalization of target genes^29,30,39^. Although, qPCR is a sensitive and efficient technique to quantify the expression profile of genes in different experimental conditions, there are several inevitable variations including mRNA quality and expression variability, identification of appropriate normalization factors becomes obligatory for accurate quantization of target genes expression profile. It becomes more imperative in comparative expression studies between different experimental conditions. To the best of our knowledge no such study has been reported in livestock species that are adapted to high altitude regions. In our study, a total of 10 candidate RGs that belonged to basic cellular processes from different functional categories were evaluated for their expression stability across high altitude adapted animals like Ladakhi Cattle, Ladakhi Yak, Double hump Camel, Ladakhi Donkey, Chanthangi Goat, Zanskar Horses. The panel of stable RGs in each livestock species were; *EEF1A1, RPL4, RPS23* (Ladakhi cattle); GAPDH, RPS9, *ACTB* (Ladakhi yak); *B2M, HPRT1, RPS23*, *ACTB* (Ladakhi donkey)*; RPS9, HPRT1 ACTB* (Changthangi goat); *HPRT1, ACTB, B2M* and *RPS23* (Double hump camel*); RPS9, RPL4, UXT* (Zanskari ponies). For different species, panel of RGs identified in PBMCs were different. Similarly, several other studies conducted by our group has also reported different panel of RGs for different experimental condition; viz., *RPS9* and *RPS15* were identified as stably expressed RGs in PBMCs of Sahiwal cows and Murrah buffaloes under heat stress conditions^22^. Similarly, both genes were also recognized as stable RGs in mammary gland of dairy cows across different stages of lactation^23^. *Beta-2M*, *RPS23*, *RPL4* and *EEF1A1* as most trustworthy RGs in heat stressed mammary explants and mammary epithelial cells of buffaloes^21,40^. *RPL4*, *EEF1A1*, *ACTB* and *GAPDH* genes were found to be most stable genes in milk derived mammary epithelial cells in Sahiwal cows during different lactation stages^41^.

In similar lines, other groups have also identified suitable RGs in different cell types or experimental conditions in livestock species. For example; *UCHL5*, *RPLP0* and *TBP* were identified as stable reference genes in whole blood samples from healthy and leukemia-virus infected cattle^42^. Interestingly, their studies, have identified *ACTB* and *GAPDH*, the two most commonly used RGs as the least stable genes. In another study, *PPIA* and *RPLP0* were identified as most appropriate RGs in milk somatic cells while *YWHAZ* was identified as most stable RG in frozen whole blood of goats infected with caprine arthritis encephalitis virus^43^. The panel of RGs were also identified in PBMCs of goat infected with peste des petits ruminants virus^44^. They found that *GAPDH* and 18S rRNA were most stable while *ACTB* was least reliable in PPRV infected PBMCs of goat. Similarly, identification of stable RGs for transcriptomic studies in bulls for meat quality trait^45^ and muscles^46^ were also reported. Tanushree et al*.*^47^ identified another panel of RGs; *GAPDH, RPS15* and *HPRT* for normalization of qPCR data in in-vitro fertilized and cloned embryos of riverine buffaloes. These studies clearly emphasized the fact that there is a need to identify panel of stably expressed genes for individual cell types, species and experimental conditions so as to achieve accurate normalization and consistency in RT-qPCR results. In the present investigation, species wise most stable RGs were identified using geNorm, Normfinder, BestKeeper and RefFinder analysis which could be quite useful in normalization of expression data in PBMC of different species adapted to high altitude environments, substantiating the importance of RGs for particular experimental conditions^30^. To the best of our knowledge, this is the first systematic attempt to identify panel of RGs across different species types adapted to high altitude hypoxia conditions.

## Conclusion

Use of reference genes or internal control genes (ICGs) or housekeeping (HKGs) genes with constant expression level between samples in response to experimental treatment or physiological state, are now considered as effective method for normalization of transcriptional data to account for the experimental variations^11^. In the present study, species wise panel of RGs were identified such as *ACTB, RPS15* in Ladakhi cattle; *GAPDH, RPS9* in Ladakhi yak; *B2M, HPRT1* in Ladakhi donkey; *HPRT1, RPS9* in Changthangi goat; *B2M, HPRT1* in Double hump camel and *RPS9, RPL4* in Zanskar ponies. To the best of our knowledge, this is the first systematic attempt to identify panel of reference genes across different livestock species adapted to high altitude region Leh-Ladakh. The data presented here could be used as a resource to select most suitable reference for accurate normalization of transcriptional data during all future studies resembling the experimental conditions highlighted in this study.

## Methods

### Livestock species, sampling and PBMCs isolation

**Figure 3:**
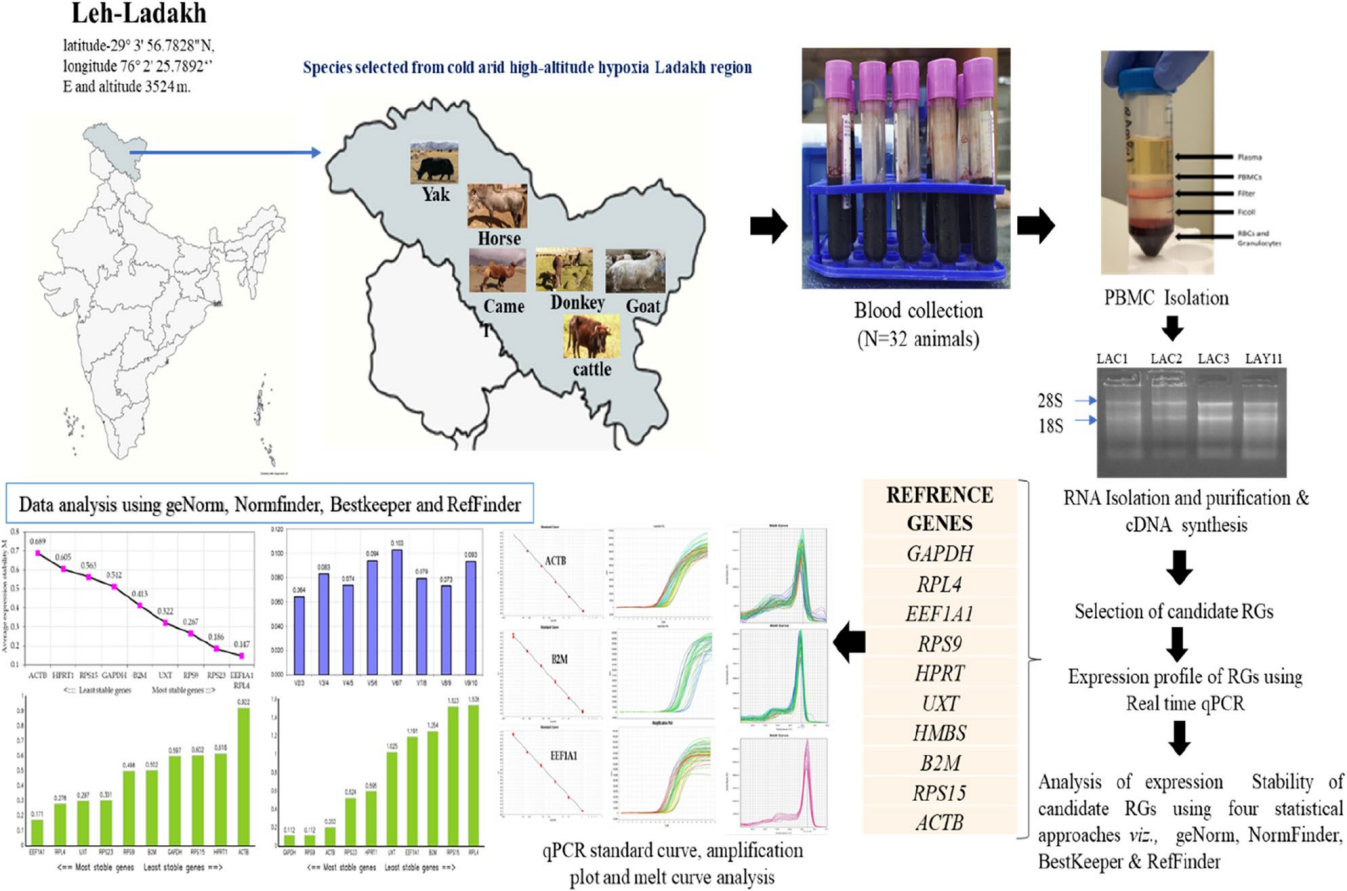
The entire workflow of the qPCR experiment conducted in PBMCs of different species adapted to cold arid hypoxia environment. Maps was made using Mapchart (https://www.mapchart.net/india.html).

The sampling was done in accordance with the guidelines and regulations of Institutional Animal Ethics Committee (IAEC). All the detailed related to animals experiment as per the ARRIVE guideline. All the procedures were approved by the animal ethics committee of ICAR-NBAGR. For this study, about 7–8 ml of blood was collected from jugular veins using EDTA vacutainer tubes from 32 individuals representing 6 native livestock species that are native of Leh-Ladakh region of India and well adapted to cold arid hypoxia conditions. For sampling, 6 young females of around 1–2 years age of each of Ladakhi cattle (LAC), Ladakhi yak (LAY) and 5 each of Ladakhi donkey (LAD), Changthangi goat (CHG), Zanskar ponies (ZAP) and Double hump camel (DHC) were randomly selected from the breeding tract of these populations. The geographical coordinates of sampling site were latitude-34° 9′ 9.3168″ N, and longitude 77° 34′ 37.3764″ E. The blood samples blood samples were transported to the laboratory for further processing and isolation of peripheral blood mononuclear cells (PBMCs). The PBMCs were isolated within 2–3 h of blood sample collection. The density gradient centrifugation procedure adopted for purification of PBMCs has been described in one of our previous publication^28^. The entire workflow of the experiment was showed in Fig. 3.

### Purification of total RNA and cDNA synthesis

For isolation of total RNA, the purified PBMCs were suspended in 1.0 ml Trizol reagent (Thermo Fisher Scientific, USA). After homogenization, the standard protocol based on chloroform and isopropanol extraction was followed to isolate the total RNA. The total RNA was further purified by employing silica-membrane RNeasy spin columns (Qiagen, Germany) along with on column digestion by DNase 1 enzyme (Qiagen, Germany) to remove traces of genomic DNA. The concentration and purity of extracted was measured using Nano view plus (Biohrome Spectros, USA). The integrity of each RNA sample was also confirmed by presence of 28S and 18S ribosomal bands on 1.5% agarose gel.

### cDNA synthesis and real time quantitative PCR (RT-qPCR)

The first strand cDNA synthesis was carried out using RevertAid First Strand cDNA Synthesis Kit (Thermo Fisher Scientific, CA, USA). First strand cDNA was synthesized using 200 ng of purified RNA, oligo-dT (18) primer, dNTP mix, random primers, RiboLock™ RNase inhibitor, M-MuLV reverse transcriptase supplied with RevertAid First Strand cDNA Synthesis kit (Thermo Scientific, CA, USA). The reaction for cDNA synthesis was set up using the program: 25 °C for 5 min, 50 °C for 60 min, and 70 °C for 15 min. The cDNA sample was diluted 1:4 (v:v) with DNase⁄RNase free water. Before subjecting for qPCR reactions, each of the cDNA samples was amplified using GAPDH in a semi-quantitative PCR. This step was done to ensure the quality of all the 32 first strand cDNA synthesized from PBMCs of 6 livestock species. The amplified products were checked on 2.5% agarose gel to ensure specific amplification. A total of 10 potential candidate RGs viz*., GAPDH, ACTB, RPS9, RPS15, RPS23, B2M, EEF1A1, RPL4, UXT* and *HPRT1* were evaluated in this study. The purpose of evaluating the stability ranking of these 10 RGs was to provide most appropriate panel of RGs in each of six livestock species of Leh-Ladakh so that any future transcriptional data could be normalized accurately. All relevant details like gene name, primer sequences, melting temperature etc. are tabulated in Table 1.

The qPCR reactions were performed in a final volume of 10 μL containing 4 μL diluted cDNA combined with 6 μL of master mix composed of 5 μL Maxima SYBR Green/ROX qPCR master mix (2X) (Fermentas Thermo, USA), 0.4 μL each of 10 μM forward and reverse primers, and 0.2 μL DNase/RNase free water. All the reactions were performed in duplicate along with six-point standard curve along with non-template control with following amplification conditions; 2 min at 50 °C, 10 min at 95 °C, 40 cycles of 15 s at 95 °C (denaturation) and 1 min at 60 °C (annealing + extension) in a Step one plus real time PCR instrument (ABI, California). For standard curve of each primer pair, fivefold serial dilution was made using pooled cDNA samples. The qPCR expression data for each gene was extracted in the form of crossing points and data was subjected for subsequent analysis.

### Identification of reference genes and statistical analysis

In order to evaluate the expression stability of RGs in individual species, 10 candidate genes viz*., GAPDH, ACTB, RPS9, RPS15, RPS23, B2M, EEF1A1, RPL4, UXT* and *HPRT1* from different functional categories were selected. four independent statistical approaches viz*.* geNorm^11^, Normfinder^48^, BestKeeper^49^ and RefFinder^50^ were used to identify most stable RGs.

The geNorm software measure the expression stability as M value which is based on overall pairwise comparison among the reference genes. The M value is inversely correlated to gene expression stability and ranks the RGs accordingly. In addition, pair wise variation analysis (V values) was also carried out using geNorm software to select optimal number of RGs to be used for normalization of target gene data. Normfinder algorithm determined the optimal RGs and the combination of two genes for a two-gene normalization factor with its corresponding stability value. The BestKeeper analysis is based on pairwise comparisons of raw cycle threshold (Ct), values of each gene. The result of BestKeeper analysis is displayed as standard deviation (SD) and coefficient of variance (CV). BestKeeper software calculated the descriptive statistics of every candidate gene and excludes the genes having standard deviation (SD) greater than 1, lower the standard deviation more is the stability of genes.

The data was analysed by direct comparing the Ct values in geNorm and Normfinder. The relative Ct values based on comparative Ct-method were the input data for geNorm and Normfinder^11,51^ wherein, the average Ct value of each duplicate reaction was converted to relative quantity data [transformed using comparative Ct method as Efficiency (^minimum Ct − sample Ct^)] with the highest expression level set to 1. As input for BestKeeper analysis, the average Ct value of each duplicate reaction was used directly (without conversion to relative quantity).

### Animal ethics

All the experimental procedure was done in accordance with the guidelines and regulations of Institutional Animal Ethics Committee (IAEC), ICAR-National bureau of animal genetic resources (ICAR-NBAGR), Karnal, Haryana, India.

## Supplementary Information

Supplementary Information.
