# Supplementary material for: Selection of species specific panel of reference genes in peripheral blood mononuclear cells of native livestock species adapted to trans-Himalayan region of Leh-Ladakh
Source: Sci Rep. 2022 Nov 2;12:18473. doi: 10.1038/s41598-022-22588-0 (PMC9630269; doi:10.1038/s41598-022-22588-0)
Supplement: Supplementary file 1 — Supplementary Information. [file 41598_2022_22588_MOESM1_ESM.pdf]

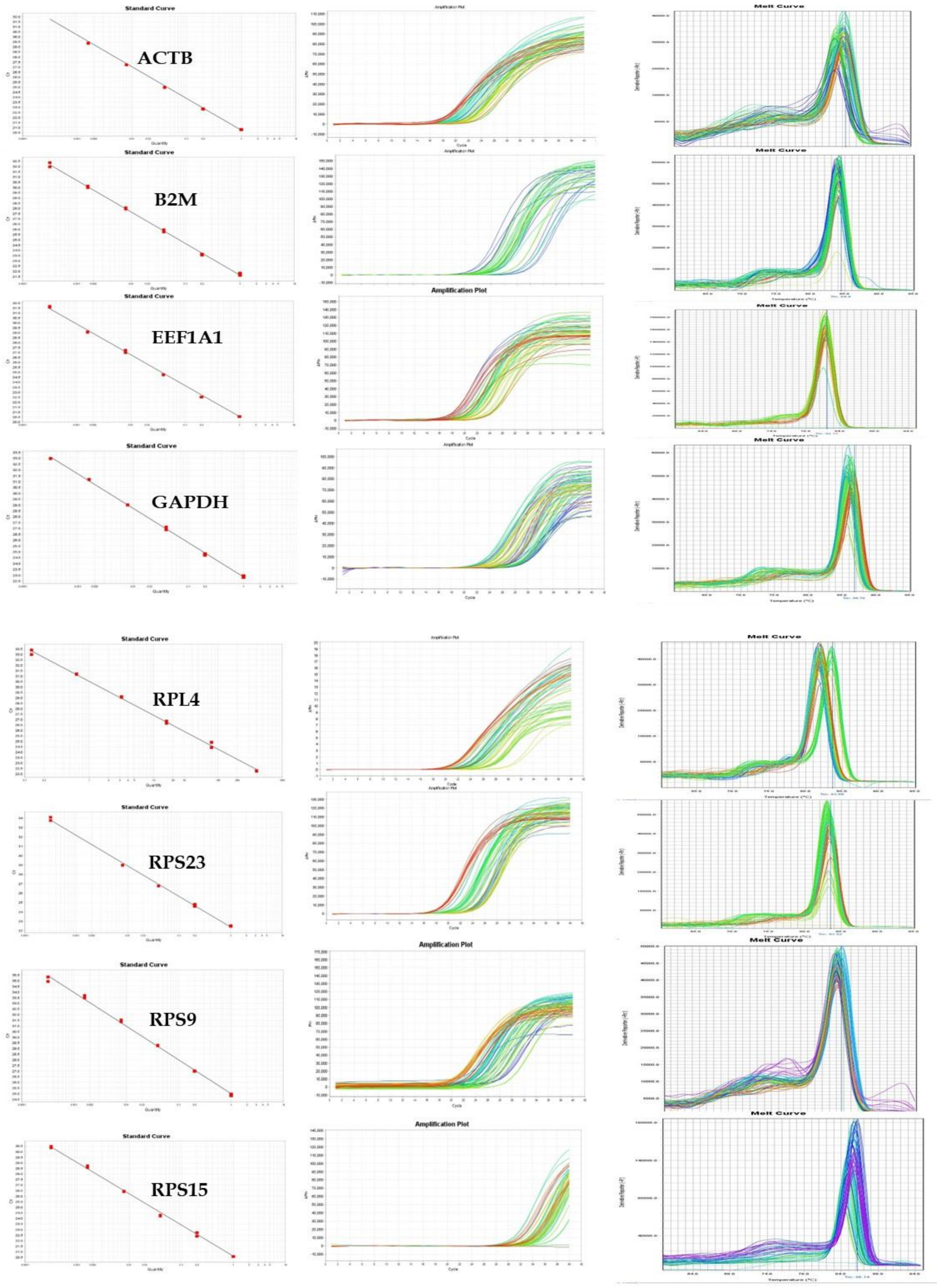
Standard Curve
Amplification Plot
Melt Curve
ACTB
B2M
EEF1A1
GAPDH
RPL4
RPS23
RPS9
RPS15

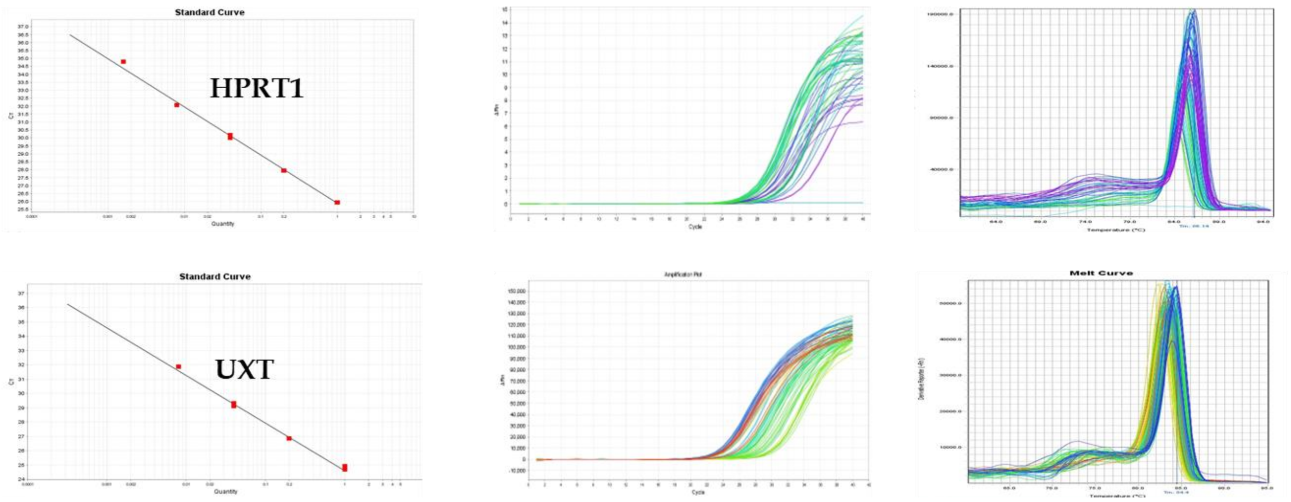

Supplementary figure S1. Standard curves, amplification plots and melting peaks for *ACTB, B2M, EEF1A1, GAPDH, RPL4, RPS23, RPS9, RPS15, HPRT1* and *UXT* RGs

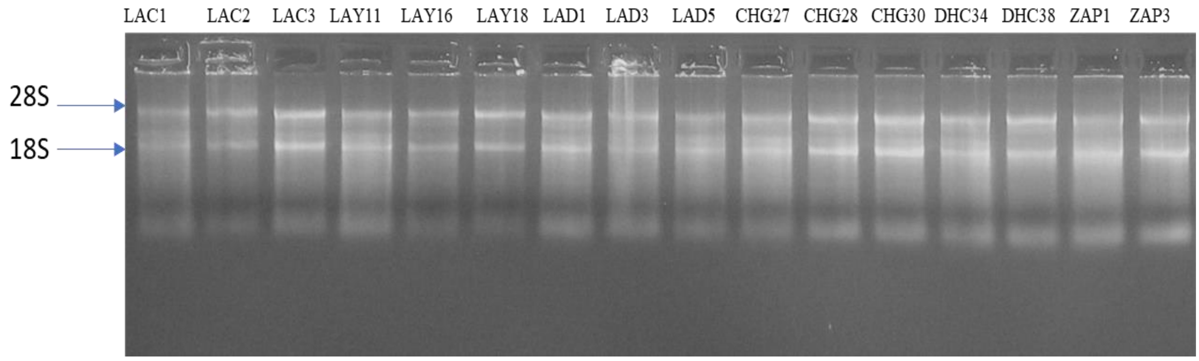

Supplementary figure S2. RNA quality corresponding to representative PBMC samples of each livestock population

Supplementary Table T1 Concentration and Purity of individual RNA samples

Animal ID	OD 260/280	Concentration
LAC1	2.11	234.6
LAC2	2.02	218.3
LAC3	2.12	327.6
LAC4	2.07	632.2
LAC5	2.05	838.3
LAC6	2.00	570.2
LAY11	2.05	144.2
LAY12	2.01	360
LAY15	2.1	185.4
LAY16	2.05	185.4
LAY18	2.1	428.6

LAY19	2.01	179.8
LAD1	2.03	176.1
LAD2	2	261.2
LAD3	2	490
LAD4	2.09	331.8
LAD5	2.1	379.6
CHG27	1.99	120.9
CHG28	2	135.2
CHG29	1.97	147.8
CHG30	2	271.2
CHG33	2.02	122.7
DHC34	1.99	184.2
DHC35	2.01	137.4
DHC37	2.07	145.4
DHC38	2.03	120.9
DHC39	2.08	275.3
ZAP1	2.06	141.4
ZAP2	2.1	154.1
ZAP3	2.08	120.7
ZAP4	2.07	188.3
ZAP5	2.04	145.3
